# Aortic Stiffness: A Major Risk Factor for Multimorbidity in the Elderly

**DOI:** 10.3390/jcm12062321

**Published:** 2023-03-16

**Authors:** Filippos Triposkiadis, Andrew Xanthopoulos, Konstantinos Lampropoulos, Alexandros Briasoulis, Pantelis Sarafidis, John Skoularigis, Harisios Boudoulas

**Affiliations:** 1 School of Medicine, European University Cyprus, 2404 Nicosia, Cyprus; 2 Department of Cardiology, University Hospital of Larissa, 41100 Larissa, Greece; 3 Department of Therapeutics, Heart Failure and Cardio-Oncology Clinic, National and Kapodistrian University of Athens, 11527 Athens, Greece; 4 Department of Nephrology, Hippokration Hospital, Aristotle University of Thessaloniki, 54642 Thessaloniki, Greece; 5 Division of Cardiovascular Medicine, The Ohio State University, Columbus, OH 43210, USA; 6 Biomedical Research Foundation, Academy of Athens, 11527 Athens, Greece

**Keywords:** multimorbidity, elderly, aortic, stiffness

## Abstract

Multimorbidity, the coexistence of multiple health conditions in an individual, has emerged as one of the greatest challenges facing health services, and this crisis is partly driven by the aging population. Aging is associated with increased aortic stiffness (AoStiff), which in turn is linked with several morbidities frequently affecting and having disastrous consequences for the elderly. These include hypertension, ischemic heart disease, heart failure, atrial fibrillation, chronic kidney disease, anemia, ischemic stroke, and dementia. Two or more of these disorders (multimorbidity) often coexist in the same elderly patient and the specific multimorbidity pattern depends on several factors including sex, ethnicity, common morbidity routes, morbidity interactions, and genomics. Regular exercise, salt restriction, statins in patients at high atherosclerotic risk, and stringent blood pressure control are interventions that delay progression of AoStiff and most likely decrease multimorbidity in the elderly.

## 1. Introduction

Multimorbidity, the coexistence of multiple health conditions in an individual [1], has emerged as one of the biggest challenges facing health services, and this crisis is partly driven by the aging population [2]. In one study including older US patients on Medicare, the prevalence of multimorbidity was 62% at ages 65 to 74 years and 82% at ages 85 years and older [3]. This problem will significantly worsen in the future. In 2020, there were an estimated 727 million people aged 65 years or over worldwide, and this number is projected to increase by 2050, reaching over 1.5 billion people [4]. 

Multimorbidity adversely affects health outcomes in older adults [5]. Further, there is compelling epidemiological evidence linking aortic stiffness (AoStiff) with several morbidities [6]. In this manuscript, following a brief summary of the structure and function of the young and aging aorta, we provide evidence linking the aging-induced increase in AoStiff to specific pathologies frequently affecting and having disastrous consequences for the elderly, such as cardiovascular disorders (hypertension, atrial fibrillation [AF], ischemic heart disease [IHD], heart failure [HF]), chronic kidney disease (CKD), anemia, chronic obstructive pulmonary disease (COPD), stroke, and dementia [7]. We conclude that increased AoStiff is a steadfast underpinning of multimorbidity in the elderly. 

## 2. The Normal Young Aorta

The aorta serves not only as a conduit, but also has important roles in affecting left ventricular (LV) performance, myocardial perfusion, central hemodynamics, and arterial function throughout the entire cardiovascular system with all these actions of the aorta globally influencing the circulation [8].

The arterial wall is made of matrix proteins (collagen fibers oriented in different directions and elastic lamellae), smooth muscle cells, and glycosaminoglycans, together with endothelial cells in the intimal layer [9]. The composition of the aortic wall changes from the central aorta towards the periphery; the media of the human ascending aorta is composed of concentric elastic lamellae (up to 60–80), interdigitated by connective tissue layers containing smooth muscle cells. This microstructure gradually fades and in medium sized, and particularly smaller vessels (such as arterioles) the number of elastic lamellae is decreased, whereas smooth muscle cells contents are increased [10].

AoStiff is the deformation resistance provided by the aorta. Due to the composition of the aortic wall, the ascending aorta is less stiff (more elastic or compliant) than the abdominal aorta (stiffness gradient) [11]. As a result, the aorta and some of the proximal large vessels act as an elastic buffering chamber in series with the LV (the Windkessel function) [12]. However, the aorta exhibits by far the largest buffering capacity in the human arterial system, with the ascending aorta and aortic arch providing approximately one-third of the total pressure buffering capacity [13].

In normal conditions, during diastole, the elastic forces of the central aortic wall forward 50% of the volume to the peripheral circulation, creating a nearly continuous peripheral blood flow. Further, pulse waves generated during the LV systole travel down the aorta to the peripheral mid-sized arteries, where they incur increased resistance due to branch points and increased arterial tone. This results in reflection of the incident waves, which subsequently travel back toward the central aorta from the periphery in phase with diastole, augmenting diastolic coronary perfusion [11].

**Figure 1:**
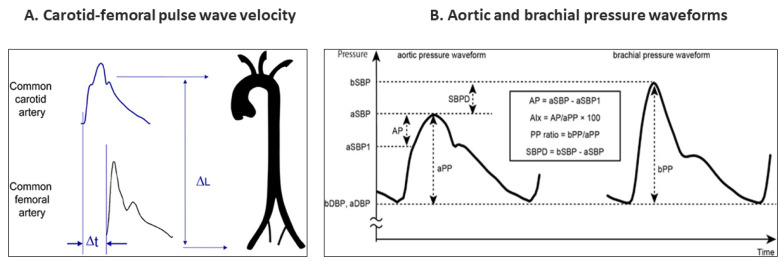
(**A**) Measurement of carotid–femoral pulse wave velocity with the foot-to-foot method. The waveforms are typically obtained transcutaneously at the right common carotid artery and the right femoral artery. The time delay (Δt, or transit time) is measured between the feet of the two waveforms. The distance (ΔL) covered by the waves is usually assimilated to the surface distance between the two recording sites, (the common carotid artery and the common femoral artery). PWV is measured as PWV = 0.8 × ΔL (m)/Δt (s). Adapted from Ref. [14] (**B**) Schematics of the aortic and brachial arterial pressure waveforms. Abbreviations: a—aortic; AIx—augmentation index; AP—augmentation pressure; aSBP1—early systolic shoulder pressure; aSBP—systolic peak pressure; b—brachial; DBP—diastolic blood pressure; PP—pulse pressure; SBP—systolic blood pressure; SBPD—SBP difference. Adapted from Ref. [16].

Evaluation of regional AoStiff is frequently conducted by measuring carotid–femoral pulse wave velocity (cfPWV), the speed at which the arterial pulse propagates along the arterial wall from the common carotid to the femoral artery (Figure 1) [13,14,15,16]. Despite its pressure dependency, cfPWV provides an average value of AoStiff and avoids the need for simultaneous measurement of pressure and area (or diameter), which is problematic for deeper arteries such as the aorta [15]. The brachial ankle PWV (baPWV) is also used. The cardio–ankle vascular index (CAVI) has been proposed as a metric that provides a “pressure-independent” assessment of arterial stiffness at the time of measurement [17]. Finally, the augmentation index (AI), which measures the degree of enhancement in the central pressure waveform due to reflected waves (Figure 1), has been reported to be associated with AoStiff and is an independent marker of cardiovascular outcomes [18].

## 3. The Aging Aorta

### 3.1. Biology

Aging is characterized by complex physiological, cellular and molecular changes leading to a progressive loss of structural and physiological integrity, organ dysfunction, and increased vulnerability to death [19]. Cellular senescence is linked with aging and presents as stable cell cycle arrest in correlation with typical morphological cellular changes and a unique secretome, the so-called senescence-associated secretory phenotype (SASP). The SASP involves pro-inflammatory factors, such as cytokines, chemokines, matrix remodeling proteases, growth factors, and lipids, of potential importance in the pathobiology of chronic diseases [20].

Classical hallmarks of aging at the molecular level have included genomic instability, loss of proteostasis, telomere attrition, deregulated nutrient sensing, epigenetic alterations, mitochondrial dysfunction, cellular senescence, and altered intercellular communication [19]. Recently, new hallmarks of aging were formulated, namely compromised autophagy, microbiome disturbance, altered mechanical properties, splicing dysregulation, and inflammation, among other emerging indicators [21].

At the level of the arterial wall, aging is associated with inflammation and oxidative stress leading to the reduced bioavailability of nitric oxide (NO), increased production of elastase and collagenase, hyperplasia, and cross-differentiation of vascular smooth muscle cells that result in functional (i.e., endothelial dysfunction) and structural changes in the arterial wall (i.e., disruption of normal elastin and collagen and substitution with altered proteins) and arterial stiffening [22].

**Figure 2:**
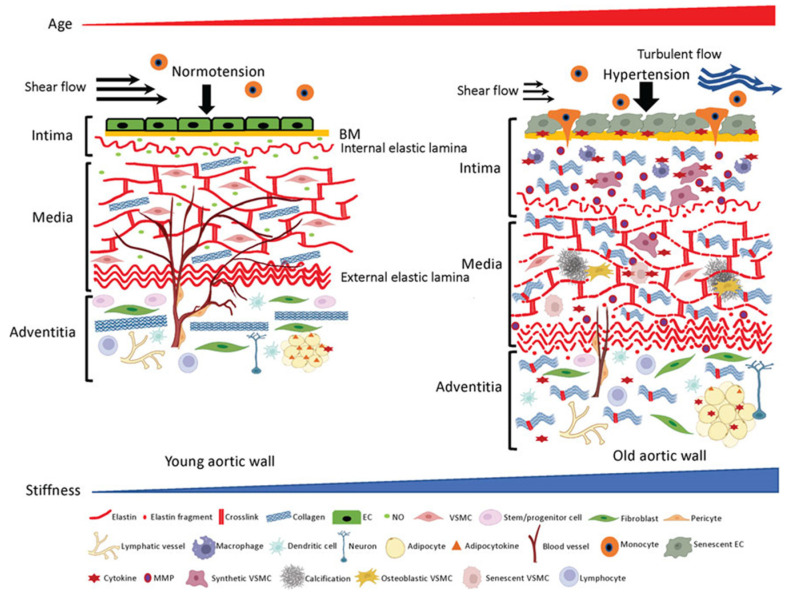
ECM structures and cellular components in the aortic wall. Young aortic wall (**left**): In intima, endothelial cells (ECs) sustain homeostasis of the wall by forming seamless barrier structure over the basement membrane (BM) and synthesizing vasoprotective factors such as nitric oxide (NO). In media, key ECM molecules (e.g., elastin, collagen) and vascular smooth muscle cells (VSMCs) form the contractile units to maintain vascular tone and compliance. In adventitia, collagen fibers reinforce the aortic wall to prevent overexpansion, and various adventitial cellular and non-cellular ECM components maintain homeostasis of the aortic wall. Aged aortic wall (**right**): Aging induces senescence of the ECs, which results in chronic low-grade inflammation and subsequent aberrant ECM remodeling (fragmentation of elastin, excess deposition of collagen and their crosslinking) in the intima and media. Adventitial fibroblasts stiffen the aortic wall by depositing excessive collagens. Adapted from Ref. [24].

Age-associated structural and functional alterations in the aortic wall, such as dilation, tortuousness, stiffening, and loss of elasticity hamper stable peripheral circulation and result in tissue and organ dysfunction in the elderly [23]. The extracellular matrix (ECM) determines the structure–function relationships of the aorta, and therefore maintains its homeostasis. ECM is pivotal for a healthy performance [24]. Age-associated remodeling of the ECM structural components, such as fragmentation of elastic fibers and excessive deposition and crosslinking of collagens, ends in functional stiffening of the aorta (Figure 2) [24].

The aging of the aorta is accelerated by several factors, including sex (pre-pubescent females have stiffer aorta than pre-pubescent males, but this difference is abrogated post-puberty), race (African Americans and Hispanic individuals may exhibit disproportionately increased AoStiff compared with Caucasians), and coexisting morbidities such as hypertension, obesity, and diabetes mellitus. Further, aortic aging is accelerated by lifestyle (e.g., excessive alcohol consumption, smoking, and physical inactivity) [25]. It should be noted, however, that in many individuals, AoStiff remains normal well after midlife, although the likelihood falls dramatically with advancing age [26], suggesting that genetic factors may also come into play.

### 3.2. Stiffness

AoStiff increases with advancing age. Invasive measurements of input impedance in humans demonstrated that AoStiff increased by 137% over the age range of 20 to 60 years [27]. Further, cfPWV increases with age, particularly after 50 years of age, when the age slope increases fourfold [28].

The aging-induced increase in AoStiff impacts blood pressure (BP) levels. Cross-sectional and longitudinal population studies have demonstrated that both peripheral systolic BP (SBP) and diastolic BP (DBP) trajectories increase progressively between adolescence and adulthood [29,30]. Before 50 years of age, the increase in both brachial DBP and SBP can be attributed to the increase in peripheral vascular resistance. DBP plateaus around the age of 50 years, and then reduces. On the contrary, SBP continues to increase even after the age of 50 in response to the continuous age-induced augmentation in AoStiff. After the age of 60 years, the different trajectories of SBP and DBP explain why PP begins to rise after age 50. The rapid widening of pulse pressure (PP) is considered to be the result of aortic stiffening. Therefore, isolated systolic hypertension is considered the most frequent subtype of hypertension after age 60 (please see below) [30].

As previously mentioned, the healthy young aorta exerts a powerful buffering function, which limits arterial pulsatility and, therefore, protects the microvasculature from potentially harmful fluctuations in pressure and blood flow. Aortic stiffening in early middle age may be a compensatory mechanism to normalize intramural wall stress [31]. However, further aortic stiffening, which occurs at late middle age and after, impairs the buffering function of the aorta, adversely affecting cardiovascular health. This is due to the elevation of SBP, which increases the LV afterload; lowering of DPB, which decreases myocardial perfusion; and abnormal ventricular–arterial interactions, which promote LV remodeling, dysfunction, and eventually failure [32]. Further, the excessive penetration of pulsatile energy resulting from the increased AoStiff into the microvasculature of organs that operate at low vascular resistance causes their damage (e.g., kidney and brain) [32]. Hypertension is also associated with aortic stiffness in animals, as demonstrated in dogs [33].

## 4. Cardiovascular Disorders

### 4.1. Hypertension

The relative timing of aortic stiffening and incident hypertension has been debated, as AoStiff is sensitive to the distending mean blood pressure (MBP) [34]. In humans, however, aortic stiffening precedes hypertension [35,36]. At the same time, high blood pressure may harm large and small arteries, resulting in further endothelial dysfunction, increased vascular stiffness, reduced lumen diameter, and formation of atherosclerotic plaques [37]. From a clinical perspective, hypertension is clearly associated with alterations in vascular function and structure that are more pronounced than the changes that would be expected as part of a normal aging process [38]. 

Regarding the pathogenetic mechanisms of hypertension in the setting of increased AoStiff, it is currently widely accepted that the increased AoStiff results in a more rapid propagation of the reflected waves from the periphery, which return in phase with cardiac systole, augmenting central SBP (systolic hypertension), increasing the hemodynamic load on the LV (and consequently on the left atrium (LA)), and decreasing the DBP, and consequently the diastolic coronary perfusion (ischemia) [25,26]. In accordance with the aforementioned points, AoStiff is an independent risk factor for major cardiovascular (CV) events in hypertensive patients. In a recent prospective cohort including 442 individuals with resistant hypertension, the changes in aortic stiffness were assessed by two cfPWV measurements performed over a median time interval of 4.7 years [39]. Patients who either increased or persisted with high cfPWV exhibited excess risks of cardiovascular morbidity/mortality compared to those who reduced or persisted with low cfPWV values. Experimental studies have demonstrated that aging and BP affect AoStiff through different mechanisms [40].

### 4.2. Atrial Fibrillation

Hypertension is considered to be the major independent risk factor for AF [41]. Hypertension, which in the elderly is predominantly due to the increased AoStiff, provokes excessive fibroblast proliferation, increased collagen accumulation, and induces cardiomyocyte apoptosis and inflammation, leading to diffused fibrosis and LV development [42]. Hypertension, in turn, further increases AoStiff with subsequent systolic and diastolic function loss, resulting in additional heart muscle remodeling. Indeed, a sub-analysis of the MESA (Multiethnic Study of Atherosclerosis) reported that PP is associated with a higher incidence of AF, even after adjusting for LA diameter and LV mass index [43]. In the same study, an association between AoStiff and new AF episodes was also observed, even when adjusting for hypertension, and this was attributed to the elevated “pulsatile load” resulting from the increased AoStiff leading to LA distension, neurohormonal activation, inflammation, and AF development. Likewise, in a cohort of 1156 individuals, AoStiff had a negative impact on LA function, independently of LV volume and function, as well as brain natriuretic peptide (BNP) levels [44]. Finally, a recent study reported that in patients with increased AoStiff, there is high risk for incident AF, and the subset of AF patients with increased AoStiff seems to be more symptomatic, while rhythm control strategies are less effective [45]. Thus, the combined influence of AoStiff and hypertension on atrial myocardial structure and electrical activity promotes AF development. 

### 4.3. Ischemic Heart Disease

AoStiff is independently associated with the progression and severity of coronary atherosclerosis [46]. In a Korean population, a CAVI >8 predicted obstructive IHD [47], whereas in a recent study that included individuals referred for coronary artery calcium (CAC) scoring and Coronary Computed Tomography Angiography (CCTA), the degree of CAC and severe coronary stenosis demonstrated significant correlation with CAVI [48]. It is noteworthy that in patients with stable angina and non-obstructive IHD, a higher aortic PWV is associated with stress-induced myocardial ischemia, and assessment of aortic stiffness was recommended for the diagnostic evaluation of this patient population [49].

The mechanisms underlying the link between AoStiff and IHD have not been delineated. Accepted explanations of the association between increased AoStif and IHD include [50,51]: (A) Increased AoStiff disturbs the oxygen demand–supply balance, causing myocardial ischemia. The LV overload due to AoStiff induced an increase in SBP and PP, resulting in LV hypertrophy and, consequently, increased oxygen demand. Further, the AoStiff-induced LV decrease in DBP is closely linked with impaired coronary perfusion. (B) AoStiff increases with age in parallel with the prevalence of traditional risk factors for IHD (e.g., hypertension, diabetes mellitus, dyslipidemia, and smoking) which also increases with age. (C) The systolic hypertension, which results from increased AoStiff, is tightly linked to endothelial dysfunction, one of the first and most important causes of the processes leading to atherosclerosis and IHD [52]. (D) Atherosclerosis underlying both IHD and increased AoStiff is characterized by the retention of lipids and inflammatory cells such as macrophages, T lymphocytes, and mast cells in the damaged arterial wall [53].

### 4.4. Heart Failure

Several studies support the association between AoStiff and incident HF. In the Framingham Heart Study, brachial PP and brachial SBP were stronger predictors than brachial DBP of congestive HF in middle-aged men and women [54,55]. Likewise, in the Multi-Ethnic Study of Atherosclerosis (MESA) the reflection magnitude (RM: ratio of backward to forward pressure wave amplitudes) was a strong and independent predictor of new-onset HF [56]. In another analysis of the Framingham Heart Study, after adjustments for standard risk factors including MBP, cfPWV was independently associated with incident HF after a follow-up of 10.1 years, although the findings did not achieve statistical significance, partly due to the modest number of HF events [57]. Further, in 2602 patients with CKD (mean glomerular filtration rate (GFR) 45 mL/min/1.73 m^2^), after a mean follow-up of 3.5 years, cfPWV was the strongest predictor of hospitalized HF [58]. Finally, in a community-based cohort of older adults, cfPWV was associated with incident HF in partially adjusted models [59], whereas in asymptomatic patients at risk of HF, an increase in brachial–ankle PWV within 5 years was associated with increased risk of incident HF [60]. It is noteworthy that an association between AoStiff assessed with CAVI and exercise capacity in patients hospitalized with decompensated HF has also been reported [61].

**Figure 3:**
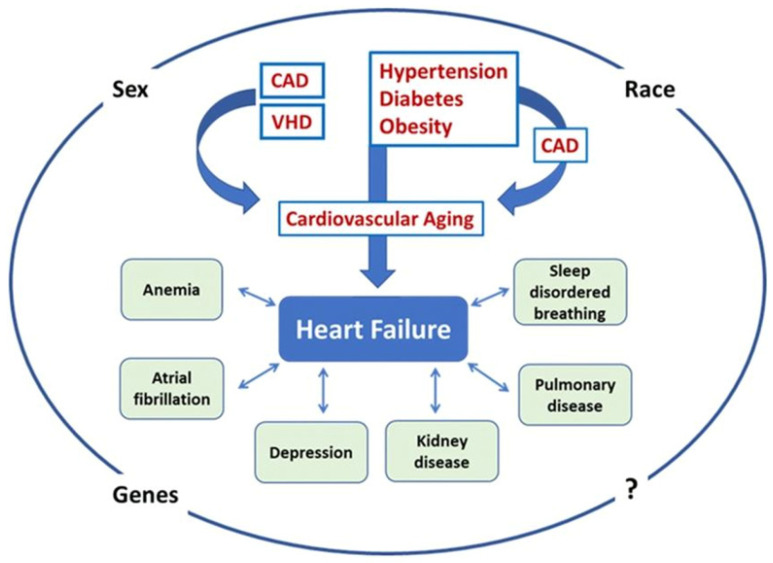
Pathogenesis of heart failure (HF) in the elderly in the Western world. HF, regardless of the left ventricular ejection fraction (LVEF), is the result of the activity of specific risk factors having effect on both the heart and the vasculature and accelerating cardiovascular aging. These risk factors (usually hypertension, obesity, diabetes, coronary artery disease [CAD], and valvular heart disease [VHD]) function individually or more commonly in groups, directly or indirectly (hypertension, obesity, and diabetes may lead to HF through an intervening myocardial infarction). The resulting HF phenotype and outcomes are additionally based on the presence and/or development of comorbidities (atrial fibrillation, anemia, depression, kidney disease, pulmonary disease, sleep disordered breathing, other) and disease modifiers (race, sex, genes, other). Adapted from Ref. [62].

HF has been attributed to the interaction between cardiovascular aging and specific risk factors, comorbidities, and disease modifiers (Figure 3) [4,62]. Wave reflections in the rigid aorta of the elderly tend to increase mid-to-late systolic LV load and myocardial wall stress [63]. When the LV pump function is preserved, the reflected wave induces a late systolic pressure peak in the pressure waveform, augmenting aortic pressure in mid-to-late systole. Conversely, when the LV pump function is depressed, wave reflection may exert more pronounced effects to decrease flow, with no apparent alteration in the appearance of the pressure waveform. In patients with severe LV systolic dysfunction, wave reflections truncate flow, reduce stroke volume and shorten the duration of LV ejection. In addition, forward waves may also be altered in patients with severely depressed LV function, as indicated by the decrease in the ratio of the first to second systolic peak, compared to individuals with preserved LV function [64].

The aging process also relates to various alterations in the heart that predispose an individual to HF [65]. These include elastin fiber degradation, increased collagen quantity, cardiomyocyte loss and hypertrophy, and endothelial dysfunction with diminished capacity to produce NO and other vital peptides. All these processes contribute to myocardial interstitial fibrosis, calcium deposition, and amyloid accumulation. Further, cardiac valves also suffer through the aging process, exacerbating cardiac stress and HF vulnerability [66].

## 5. Chronic Kidney Disease

The kidneys are adversely affected by the increased AoStiff because they are high-flow, low resistance organs receiving approximately 20% of the resting cardiac output. Due to the anatomy of renal microcirculation with small number of branches between the aorta and the glomeruli, the main protective mechanism against increased transmission of pulsatile pressure to the glomerulus is the myogenic response of the afferent arteriole, which can normally autoregulate renal blood flow within a wide range of preglomerular perfusion pressures, in co-ordination with the tubulo-glomerular feedback mechanism [67]. In a rigid aorta with lost “windkessel effect” a greater percentage of pulsatile power in the arterial pressure and flow waveforms penetrates deeper into the renal microcirculation, where it may cause microvascular damage in the preglomerular vessels [68]. In people with compromised renal blood flow autoregulation (i.e., in the elderly or patients with diabetes mellitus) [69,70], this arterial stiffness-related pressure pulsatility is also transmitted to a greater extent in the glomerular capillary. As such, it can be a major contributor to glomerular hypertension and hyperfiltration, and the development of typical albuminuric chronic kidney disease [71].

The association between AoStiff and renal dysfunction has been repeatedly demonstrated. Out of the 3666 participants (mean age = 65 years old; 58% women) from the Rotterdam Study, 601 participants with incident CKD were recognized during a median follow-up of 11 years [72]. In the model adjusted for age, sex, mean arterial pressure, heart rate, and baseline GFR, each standard deviation (SD) higher PP was associated with 0.15-mL/min per 1.73 m^2^ steeper annual eGFR decline (95% confidence interval [95% CI], 0.10 to 0.20) and 11% higher risk of incident CKD (95% CI, 1.05 to 1.18), whereas each SD higher PWV was associated with 7% higher risk of incident CKD (95% CI, 1.00 to 1.14). Similar findings of a prospective study [73] including individuals aged ≥40 years without overt kidney disease reported that PWV, age, and eGFR were independent predictors of renal function decline. In the same study, baseline eGFR did not determine the annual change in PWV, suggesting a unidirectional association between arterial AoStiff and eGFR.

At this point, it is critical to note that kidney aging is a complex phenomenon, which is often difficult to distinguish from CKD. The volumes of the kidney cortex and medulla do not alter in parallel with healthy aging, as up to 50 years of age, cortex volume decreases, whereas medulla volume increases. Microstructural features of healthy aging include nephron loss, increase in the glomeruli (predominantly of the superficial cortex) which are globally sclerosed and ischemic, and a higher density of small interstitial fibrosis and tubular atrophy foci [74].With unhealthy aging, morbidities such as obesity and diabetes both increase AoStiff, accelerate microstructural pathology observed in aging, and contribute to disease-specific pathology, such as the solidification form of glomerulosclerosis.

AoStiff increases both renal injury progression and mortality in patients with established CKD, independent of BP. The CRIC (Chronic Renal Insufficiency Cohort) study examined the relationship between cfPWV end-stage kidney disease (ESRD), ESRD or halving of eGFR, or death from any cause in 2795 participants (mean age of 60 years, 56.4% were men, average eGFR at entry was 44.4 mL/min per 1.73 m^2^) [75]. During follow-up (4.9+/−2.1 years) patients in the highest tertile of PWV (>10.3 m/s) were at higher risk of ESRD, ESRD or 50% decline in eGFR, or death.

**Figure 4:**
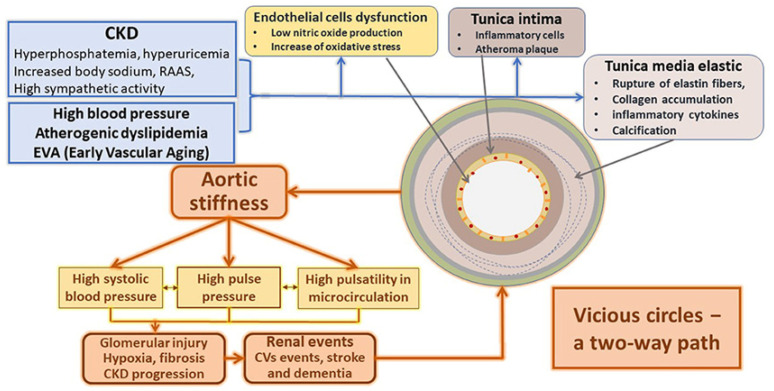
Main mechanisms responsible for the structural and functional changes in the arteries due to CKD. Adapted from Ref. [76].

Conversely, severe CKD is associated with a dramatic increase in AoStiff (Figure 4) [76]. Severe CKD is associated with mineral and bone disorders (CKD-MBD). The key players of CKD-MBD, calcium, phosphate, parathormone (PTH), fibroblast growth factor 23 (FGF23), and the vitamin D hormonal system are adversely affected by the deterioration of kidney function, which deregulates the tightly interrelated mechanisms that control these parameters [77]. As a result, important changes occur in the bone and mineral hormonal axis, leading to disrupted bone turnover and adversely affecting clinical outcomes, such as decrease in bone mass associated with increased bone fragility and bone fractures and increased valvular and vascular calcifications, which greatly impact cardiovascular outcomes. A study including patients in CKD stage 5D reported that vascular calcifications are frequently located in the aorta (80%) and the coronary arteries (about 60–70%), and less frequently in small-caliber arteries (20–30%) [78]. The time (years) spent on hemodialysis has been positively associated with vascular calcifications, with each year on dialysis increasing the risk of developing vascular calcification by 15% [79]. Consequently, similarly to pre-dialysis CKD patients, several longitudinal studies in CKD stage 5D have shown that underlying AoStiff insensitive to BP changes is the most prominent risk factor for cardiovascular outcomes in this population [80,81,82,83].

## 6. Anemia

Anemia is quite commonly diagnosed in older adults and is an important indicator of several reactive and clonal conditions. In a retrospective study of more than 19,000 hospital patients, the incidence of anemia increased from 15% at the ages of 64–69 to 37% in those over aged 90 [84]. The etiology of anemia in the elderly is multifactorial and ranges from bone marrow failure syndromes to CKD and HF, and from nutritional deficiencies to inflammatory processes, including inflammaging in immunosenescence. In several cases, though, no clear-cut etiology is identified. These patients are referred to as unexplained anemia in the elderly (UAE) [85].

The mechanisms linking AoStiff with UAE are diverse and complex. There is evidence to suggest that AoStiff is inversely associated with red blood cell volume [86] and erythropoietin (EPO) levels [87] in healthy individuals. It is reasonable, therefore, to assume that AoStiff is a contributing factor to the typical low EPO concentration observed in UAE, impairing the endocrine feedback pathways governing the basal regulation of kidney EPO synthesis, as well as renal perfusion. In this regard, it is plausible that an increase in AoStiff may lead to baroreceptor dysfunction, hindering the release of blood volume-regulating hormones that directly stimulate EPO synthesis [88]. Further, increased AoStiff is strongly associated with a reduction in renal perfusion contributing to impaired hormone delivery to EPO-producing cells. Admittedly, however, the potential impact of AoStiff on UAE etiology deserves further investigation.

## 7. Ischemic Stroke

Ischemic stroke may result from a focal occlusion or stenosis of an artery or multiple arteries in the brain (intracranial occlusion) or leading to the brain (extracranial cervical artery occlusion). These focal occlusions develop due to several mechanisms, including cardioembolism, artery-to-artery thrombo-embolism, occlusive arterial disease, and cerebral small vessel disease (CSVD) [89]. Lacunar strokes result most commonly from small vessel mechanisms, but alternative mechanisms are feasible.

There is compelling evidence of a positive correlation between AoStiff and ischemic stroke. In the seminal study of Laurent et al., which included 1715 essential hypertensive patients (mean follow-up 7.9 years) there was a relative risk (RR) increase of 1.72 (95% CI, 1.48 to 1.96; *p* < 0.0001) for each standard deviation (SD) increase in cfPWV (4 m/s), which remained significant after adjustment for classic cardiovascular risk factors [90]. These findings were subsequently confirmed in several studies. In a meta-analysis including 17,635 participants, a total of 1785 (10%) had a cardiovascular disease (CVD) event. The pooled age- and sex-adjusted hazard ratios (HRs) per 1-SD change in log_e_ aortic PWV for stroke was 1.54 (95% CI: 1.34 to 1.78; *p* < 0.001) and remained significant (HR: 1.28 [95% CI: 1.16 to 1.42]; *p* < 0.001) after adjusting for conventional risk factors [91]. It is noteworthy that an association between increased cfPWV and cerebral artery calcification or stenosis has been reported in hypertensive subjects [92], as well as in patients with acute ischemic stroke [93].

The association between AoStiff and ischemic stroke has been attributed to several mechanisms which are not mutually exclusive. Hemodynamic alterations secondary to AoStiff may come into play. Raised PP promotes arterial remodeling, increases arterial wall thickness, and induces the development of atherosclerotic plaques, which may rupture or ulcerate leading to intravascular thrombosis [94]. Further, the rigid aorta facilitates the penetration of excessive pulsatile energy into the cerebral microcirculation, resulting in its damage [95]. Undoubtedly, however, the fact that the measured high aortic AoStiff may reflect parallel structural changes in the intracerebral vasculature, such as breaking of elastic fibers, fibrosis, calcifications, medial smooth muscle necrosis, and diffusion of macromolecules into the arterial wall, cannot be excluded [96]. Finally, the classical cardiovascular risk factors and diseases (e.g., hypertension, atherosclerosis, coronary artery disease, and CKD), which are promoted by AoStiff, are also risk factors for ischemic stroke [97].

## 8. Dementia

Neurodegenerative dementias such as Alzheimer’s disease are the most frequent, followed by microangiopathies such as vascular dementia (VaD) and Lewy body dementia (LBD), with mixed pathology often seen. 

Dementia is a syndrome defined by the deterioration of cognitive function beyond that expected as a result of biological aging, which presents as a disturbance of multiple higher cortical functions including learning and memory, complex attention, executive function, language, motor perception, and social cognition, affecting the patient’s ability to perform daily activities independently [98]. Dementia is an umbrella term for a range of conditions characterized by progressive cognitive impairment and behavioral changes that interact with daily functioning [99,100].

Different diagnostic criteria have been proposed for VaD, all consisting of the same three core elements: established (1) acquired cognitive impairment, (2) vascular damage in the brain, and (3) a causal association between the two [101]. Factors characterizing subtypes of VaD encompass the nature and extent of vascular pathologies, the degree of involvement of extra and intracranial vessels, and the anatomical location of tissue changes, as well as the time after the initial vascular event. Cerebral small vessel disease (CSVD), characterized by arteriolosclerosis, lacunar infarcts and cortical and subcortical microinfarcts, and diffuse white matter changes, which involve myelin loss and axonal abnormalities, has gained prominence as an important substrate of cognitive impairment [102]. 

**Figure 5:**
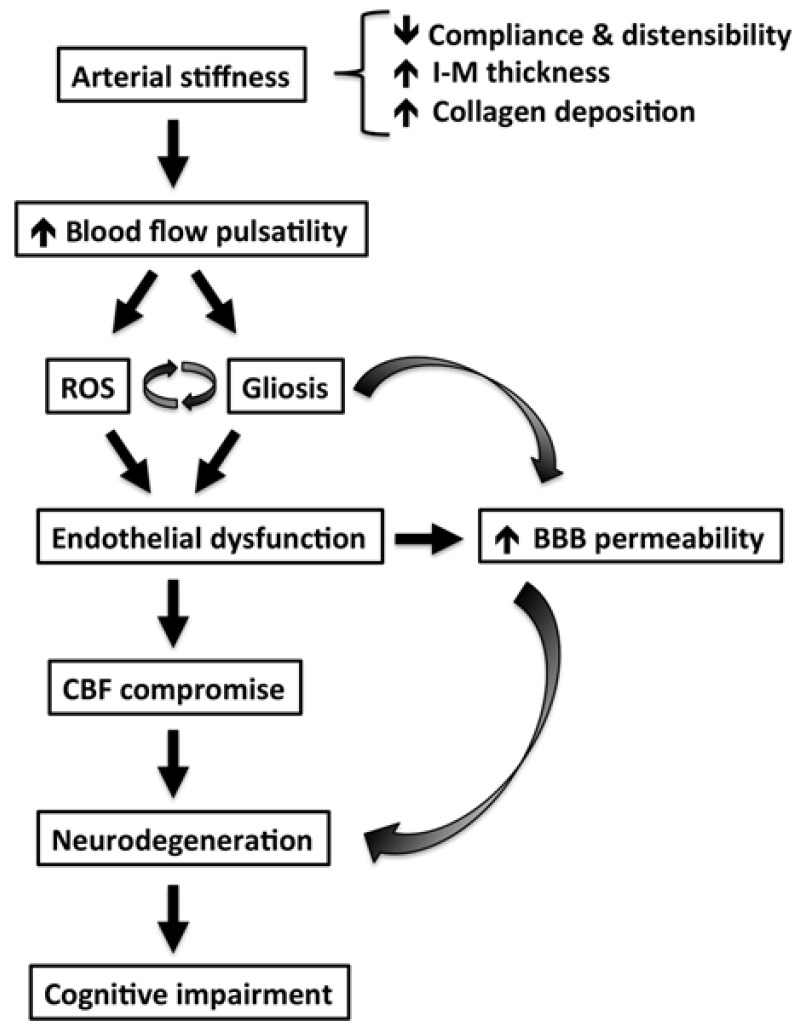
Proposed mechanisms by which arterial stiffness leads to cognitive impairment and brain dysfunction based on evidence from animal studies. Abbreviations: IM—intima-media; ROS—reactive oxygen species; BBB—blood–brain barrier; CBF—cerebral blood flow. Adapted from Ref. [103].

The brain, like the kidney, is a high-flow organ receiving approximately 20% of resting cardiac output [68]. Increased AoStiff exposes the small vessels in the brain to abnormal flow pulsations and, as such, may contribute to the pathogenesis of CSVD and cognitive impairment (Figure 5) [103].

In a population-based study, the association between AoStiff, as measured by cfPWV and small vessel disease, was investigated in 1460 participants who underwent brain magnetic resonance imaging (MRI) scanning. A higher cfPWV was associated with a larger white matter lesion volume, but not with lacunar infarcts or microbleeds [104]. However, in individuals with uncontrolled hypertension, a higher aPWV was significantly associated with larger white matter lesion volume, deep or infratentorial microbleeds, and, to a lesser extent, with lacunar infarcts. In the same direction were the findings of another study, which included 953 participants who underwent baPWV and brain magnetic MRI. Increased AoStiff was associated with most of the imaging markers of CSVD, including dilated perivascular spaces in white matter, larger white matter hyperitensity volume, strictly lobar microbleeds, and brain atrophy, but not lacunes [105]. It has been suggested that the hemodynamic stress, pulsatile pressure, and blood pressure variability resulting from the increased AoStiff can cause a ‘tsunami effect’ towards the cerebral parenchyma and lead to cerebral small vessel disease [106]. 

In addition to its contribution to the development of CSVD, AoStiff is strongly linked with the progressive deposition of Aβ in the brain in the elderly, indicating a correlation between the severity of subclinical vascular disease and progressive cerebral Aβ deposition [107]. Autopsy and amyloid positron emission tomography (PET) studies established the existence of comorbid CVD and amyloid deposition in patients with cognitive impairment. Underlying microvasculature changes may contribute to an early accumulation of amyloid. With aging, the emergence of CVD could further impair cognition in those individuals with preexisting amyloid by causing direct neuronal damage and accelerating the amyloid deposition. Concurrent CVD may also affect the brain’s reserve against cognitive effects from amyloid disposition, and vice versa [108]. 

The Atherosclerosis Risk in Communities (ARIC)-Neurocognitive Study collected detailed cognitive testing for adjudication of dementia and mild cognitive impairment (MCI), brain MRI, and arterial stiffness by cfPWV and heart–carotid PWV (hcPWV). The ARIC-PET ancillary study added Aβ imaging using florbetapir ([18F]-AV-45) to obtain standardized uptake volume ratios and define global Aβ-positivity. AoStiff, measured by PWV proved a risk factor for dementia through its repeated relationships with cognition, CSVD, and Aβ deposition [109].

## 9. Frailty

Frailty is defined as increased vulnerability to poor resolution of homoeostasis after a stressor event (i.e., infection, surgery, or new medical treatment), which increases the risk of adverse outcomes (i.e., death or hospitalization), and it may present as unexplained weight loss, severe fatigue, falls, delirium, or fluctuating disability (i.e., “good and bad days”) [110]. Its prevalence increases with ageing and has been reported to range between 12–24% among people aged ≥50 across 62 countries [111]. Frailty and CVD share common risk factors, namely hypertension, diabetes mellitus, and obesity [112]. The potential common underlying pathophysiologic mechanism is inflammation (“inflammageing”) [112,113]. Arterial factors, especially AoStiff, have been suggested to be a potential linking mechanism between frailty and CVD. Frailty has been associated with an increased risk of unfavorable outcomes in patients with peripheral artery disease [114], valvular disease [115,116], as well as in adults with [117] and without coronary artery disease [118]. A recent systematic review and meta-analysis of five cross-sectional studies and 7575 participants, aged above 60 years, revealed an association between frailty (defined as a clinical syndrome in which three or more of the following criteria were present: (a) unintentional weight loss (10 lbs in past year), (b) self-reported exhaustion, (c) weakness, (d) slow walking speed, and (e) low physical activity or defined as Simple Frailty score = 2) and prefrailty (defined as Simple Frailty score = 1) and greater AoStiff, compared to non-frail patients [119]. Another systematic review and meta-analysis of 29 cross-sectional and 9 longitudinal studies (43,115 participants) demonstrated an independent association between arterial stiffness, measured using PWV, and cognition (a component of frailty) [120]. Lastly, in the community-dwelling elderly population of the Wakayama Study, the loss of skeletal muscle mass and function was associated with increased arterial stiffness, expressed as baPWV [121].

## 10. Degenerative Aortic Stenosis

Degenerative aortic valve stenosis (AVS), the most frequent cause of interventional valve treatment, is characterized by increased LV afterload. The LV load is determined not only by the degree of valve stenosis, but also by the arterial stiffness [122]. Therefore, unsurprisingly, increased arterial stiffness has been associated with unfavorable outcomes in patients with AVS. For example, Tanaka et al. examined the correlation between pre-procedural baPWV and 1-year post- Transcatheter Aortic Valve Implantation (TAVI) all-cause death and HF rehospitalization in a cohort of 161 patients with severe degenerative AVS [123]. The authors reported a significantly higher cumulative 1-year composite outcome in the high baPWV group (i.e., >1639 cm/s) compared to the low (i.e., <1639 cm/s) baPWV group (31% vs. 10%; log-rank test, *p* < 0.001), whereas high baPWV was an independent predictor of the 1-year combined outcome (adjusted HR, 3.42; 95% CI, 1.62–7.85; *p* = 0.002). Broyd et al., utilizing data from 186 patients who underwent TAVI between April 2016 and June 2017, showed that PWV higher than 11.01 m/s was the only predictor of 1-year mortality following TAVI (Odds Ratio 3.57, 95% CI 1.36–9.42, *p* = 0.01) [124]. An elegant study demonstrated that increased vascular stiffness (i.e., Resistive index ≥ 0.7 and pulsatile index ≥ 1.3) was associated with the combined outcome of cardiovascular death and HF rehospitalization, during 3-year follow up, in 246 consecutive patients with severe symptomatic degenerative AVS (aortic valve area, AVA < 1.0 cm^2^) referred for surgical (AV replacement) or interventional treatment (TAVI) [125]. Future studies are needed to reveal the ideal non-invasive method to assess arterial stiffness in AVS patients and establish the prognostic value of the arterial stiffness alterations after AV replacement [122].

## 11. Determinants of Multimorbidity Pattern in the Elderly

The stiff aorta and the tightly linked hypertension contribute to the development of several morbidities in the elderly that appear in groups of two or more (multimorbidity) and adversely affect outcomes. Recognition of specific patterns will help clinicians predict the possible occurrence of multimorbidity risks among patients and prevent or intervene those risks both at AoStiff and at group level. The factors that determine the development of specific patterns include:

**1. Sex and ethnicity.** Women are more often affected by multimorbidity than men. In the Rotterdam study (*n* = 6094, median years of follow-up 9.2) two-thirds of people over 45 developed multimorbidity in their remaining lifetime, with women manifesting nearly double the risk of multimorbidity compared to men [126]. MicroRNAs (miRNAs) are small, non-coding endogenous RNA molecules that regulate gene expression either by mRNA cleavage/destabilization or inhibition of translation. miR-181a, which acts as an inflamma-miRNA playing important roles in the aging process, has been identified as multimorbidity-associated miRNA [127].The results of a recent study highlighted sex specific correlations of miR-181a with risk factors for negative outcomes, suggesting that the routes by which this miRNA can affect health status are not the same between sexes [128].

In addition to sex, contextual features (ethnic characteristics, living habits, etc.) contribute to the development of diverse multimorbidity patterns in different regions and countries. A study analyzed data from the Collaborative Research on Aging in Europe project (Finland, Poland, and Spain) and the World Health Organization’s Study on Global Ageing and Adult Health (China, Ghana, India, Mexico, Russia, and South Africa) including information from 41,909 noninstitutionalized adults older than 50 years [129]. Overall multimorbidity prevalence was elevated across countries. Russia had the highest prevalence of multimorbidity (71.9%), whereas China (45.1%) and Ghana (48.3%) had the lowest. The most prevalent comorbid condition was hypertension, especially among those with obesity, stroke, diabetes, and angina.

**2. Common pathogenic morbidity routes.** In aging populations many patients have multiple diseases characterized by acceleration of the normal aging process. Common signaling pathways and cellular events have been identified in the pathogenesis of increased AoStiff, CVD, CKD, and neurodegenerative disease. These include cellular senescence with telomere shortening, activation of PI3K–AKT–mTOR signaling, impaired autophagy, mitochondrial dysfunction, stem cell exhaustion, epigenetic changes, abnormal microRNA profiles, immunosenescence, and low-grade chronic inflammation (“inflammaging”). Many of these pathways are driven by chronic oxidative stress. There is also a reduction in anti-aging molecules, such as sirtuins and Klotho, which further accelerates the aging process [130,131,132].

**Figure 6:**
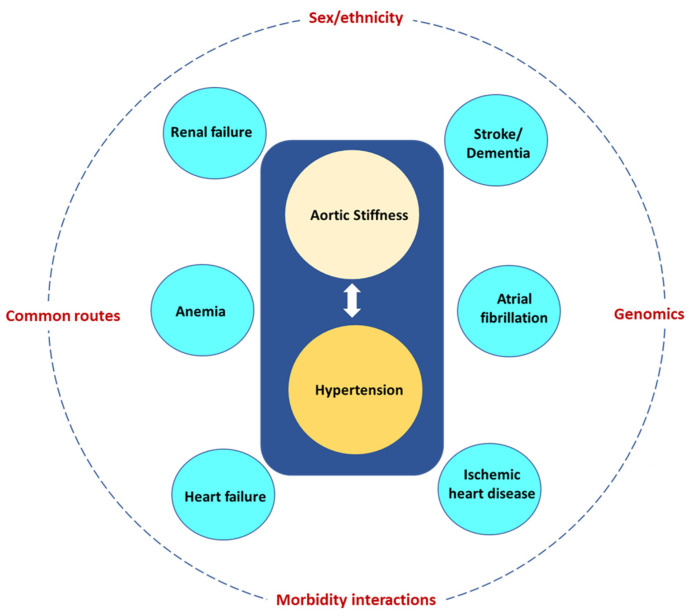
Aortic stiffness and the tightly linked hypertension give rise to several morbidities (blue circles) most of them being powerful risk factors for other morbidities. The eventual multimorbidity pattern depends on modifiers, such as sex and ethnicity, common routes, morbidity interactions, and genomics.

**3. Morbidity interactions.** Increased AoStiff and the tightly linked hypertension give rise to morbidities, which in turn lead to other morbidities and further increase AoStiff. A common scenario is illustrated in Figure 6. Increased AoStiff causes hypertension, which in turn further increases AoStiff and may give rise to HF and renal failure, further increasing AoStiff and the severity of hypertension and leading to a vicious cycle. To complicate things further, hypertension is a powerful risk factor for AF, IHD, stroke, and dementia, whereas both renal failure and HF are powerful risk factors for the development of anemia. The above considerations are in accordance with a prospective analysis using the data from the Survey of Health, Ageing and Retirement in Europe in both 2013 and 2015 [133]. Approximately 380 unique combinations of chronic disease were identified in older adults with multimorbidity, with hypertension existing in almost every prevalent disease combination. Similar findings were detailed in another study in Ireland, which reported that hypertension and hypercholesterolemia were the most common co-existing diseases. This was determined by analyzing the chronic diseases combinations from 6101 old adults aged 50+ [134]. Finally, the high prevalence of hypertension in multimorbidity patterns was confirmed in a study including 7480 individuals aged 60+ in Shanxi province, China [135].

**4. Genomics.** Genetic data can be used to identify genetic overlaps between multiple diseases, including those that might not be known to have shared genetic pathways [136]. Using the results of published GWAS (genome wide associated studies), Melzer and colleagues uncovered 22 genetic variants that were linked with multiple age-related diseases, 12 of which were linked and mostly inherited together [137]. One of these twelve genetic variants was found in apolipoprotein E (APOE) and was linked with Alzheimer’s and coronary artery disease, whereas two other DNA variants were located in the genes LPA (lipoprotein a) and LDLR (low-density lipoprotein receptor) and were linked to altered blood lipids and cardiovascular traits. The remaining nine associations between genetic variants that tended to be inherited together and age-associated diseases included links to three or more of the diseases, including Alzheimer’s disease, stroke, coronary artery disease, type 2 diabetes, kidney disease, osteoarthritis, and several cancers. The phenomenon in which a single genetic variant or gene affects multiple biological pathways or diseases is called “pleiotropy”. Likewise, another study reported an atlas of 11,285 multimorbid disease pairs among 438 common diseases, with 46% of the multimorbidities with available genetic information sharing genetic components in at least one of the three levels—loci, network, or overall genetic architecture [138].

Another way to illustrate the effect of genetics in multimorbidity is by using polygenic risk scores (PRS), which are estimates of individuals’ genetic liability to a trait or disease and are calculated according to their genotype profile and relevant GWAS data [139]. A study including 198,965 dementia-free participants aged ≥60 used a PRS based on 38 non-apolipoprotein E single-nucleotide polymorphisms and APOE ε4 status to determine the genetic risk for dementia [140]. Over 15 years of follow-up, 6270 participants developed dementia and hypertension was associated with a 19% increased risk of dementia (HR = 1.19, 95% CI 1.11–1.27). The associations remained similar when stratifying by genetic risk, with no evidence for multiplicative interaction by dementia PRS or APOEε4 status. However, the risk difference between those with and without hypertension was larger among those at higher genetic risk.

## 12. Clinical Implications

Increased AoStiff is a major risk factor for the development of multimorbidity in the elderly, and its reduction may potentially provide substantial health benefits [141]. In this regard, several lifestyle and pharmacological interventions have already proved to be effective in preventing or ameliorating arteriosclerosis and increased AoStiff associated with aging [142]. A typical example is salt restriction. A recent systematic review and meta-analysis of randomized control trials including 14 cohorts with 431 participants and 1–6 weeks intervention time demonstrated that an average reduction in sodium intake of 89.3 mmol/day was associated with a 2.84% (95% CI: 0.51–5.08) reduction in cfPWV [143]. Furthermore, weight loss in normotensive overweight/obese young adults has been related to reduced cfPWV independently of changes in established hemodynamic (blood pressure) and cardiometabolic risk factors [144].

Regular exercise (aerobic or resistance) has been linked to several health benefits, including improvements in functional capacity, traditional risk factors, and cognitive function. An interesting study in 146 healthy individuals from the Baltimore–Washington area revealed 26% lower cfPWV in the subgroup of endurance-trained male athletes aged 54 to 75 years old, compared to their age- and blood pressure-matched sedentary peers [145]. On top of this observation, habitual physical activity has been reported to attenuate large artery stiffness due to aging [146,147]. Aerobic exercise may improve arterial stiffness via a reduction in sympathetic nerve activity, improving nitric oxide (NO) bioavailability, as well as exerting anti-inflammatory actions [148]. On the contrary, two recent meta-analyses found no association between resistance exercise and large artery stiffness in healthy individuals or those at risk of cardiovascular disease [149,150].

Pharmacological agents such as angiotensin converting enzyme (ACE) inhibitors, β-blockers, aldosterone antagonists, sodium–glucose co-transporter-2 (SGLT-2) inhibitors and nitrates may reduce arterial stiffness independent of their action in heart rate or blood pressure [151,152]. Nevertheless, when a pharmacological agent reduces mean blood pressure, it lowers arterial stiffness as well [32]. In this regard, a recent post hoc analysis of the STEP trial (Strategy of Blood Pressure Intervention in the Elderly Hypertensive Patients) confirmed that arterial stiffening precedes SBP in both the intensive and standard treatment groups, and it led to difficulty in reaching target SBP, particularly in the intensive treatment group [153]. In the same study, assignment to the intensive treatment group was associated with an attenuation of the age-related increase in arterial stiffness assessed with the baPWV at 3-year follow-up. Finally, a recent study including 5105 adults with high atherosclerotic risk demonstrated an association between statin use and slower progression of AoStiff assessed with baPWV [154].

## 13. Conclusions

AoStiff contributes to the development of pathologies frequently affecting the elderly, such as cardiovascular disorders (hypertension, AF, IHD, HF, CKD, anemia, stroke, and dementia). The coexistence of the aforementioned morbidities in groups of two or more (multimorbidity) is very frequent in the elderly. The appearance of specific multimorbidity patterns depends on several factors, including sex, ethnicity, common morbidity pathogenic routes, morbidity interactions, and genomics. Hypertension is probably the most important morbidity, as it further increases AoStiff, is causally related to most of the other pathologies, and is present in most multimorbidity patterns. Prevention and treatment of increased AoStiff may be one of the most important strategies for the management of multimorbidity in the elderly.

## Data Availability

Not applicable.
